# A Porous π-Stacked Self-Assembly of Cup-Shaped Palladium Complex for Iodine Capture

**DOI:** 10.3390/molecules28072881

**Published:** 2023-03-23

**Authors:** Lin-Lin Li, Min Huang, Ting Chen, Xiao-Feng Xu, Zhu Zhuo, Wei Wang, You-Gui Huang

**Affiliations:** 1 College of Chemistry, Fuzhou University, Fuzhou 350108, China; 2 CAS Key Laboratory of Design and Assembly of Functional Nanostructures, and Fujian Provincial Key Laboratory of Nanomaterials, Fujian Institute of Research on the Structure of Matter, Chinese Academy of Sciences, Fuzhou 350002, China; 3 Xiamen Institute of Rare Earth Materials, Haixi Institutes, Chinese Academy of Sciences, Xiamen 361021, China; 4 College of Chemistry and Materials Science, Fujian Normal University, Fuzhou 350002, China; 5 Fujian Science & Technology Innovation Laboratory for Optoelectronic Information of China, Fuzhou 350108, China

**Keywords:** nuclear waste, iodine, porous, *π*···*π* stacking, cup-shaped

## Abstract

Acquiring adsorbents capable of effective radioiodine capture is important for nuclear waste treatment; however, it remains a challenge to develop porous materials with high and reversible iodine capture. Herein, we report a porous self-assembly constructed by a cup-shaped Pd^II^ complex through intermolecular *π*···*π* interactions. This self-assembly features a cubic structure with channels along all three Cartesian coordinates, which enables it to efficiently capture iodine with an adsorption capacity of 0.60 g g^−1^ for dissolved iodine and 1.81 g g^−1^ for iodine vapor. Furthermore, the iodine adsorbed within the channels can be readily released upon immersing the bound solid in CH_2_Cl_2_, which allows the recycling of the adsorbent. This work develops a new porous molecular material promising for practical iodine adsorption.

## 1. Introduction

As a non-greenhouse energy source, nuclear energy is most likely to replace traditional fossil fuels [1,2]. Currently, nuclear energy is widely applied in many areas related to human life [3]. With the rapid development of the nuclear energy industry, the safe disposal of nuclear waste containing radioactive species, especially radioactive iodine, has become a significant concern [4,5,6,7,8]. Both ^129^I and ^131^I, which are the main radioisotopes for iodine, are harmful to its ecological surroundings and human health. ^129^I is extremely dangerous because it has a long half-life (1.57 × 10^7^ years) and can be accumulated in the human thyroid gland, causing serious diseases [6]. As for ^131^I, it is often combined with hydrocarbons, giving rise to harmful organic compounds such as methane iodide [9,10,11,12]. Among various possible radioactive iodine species, molecular iodine (I_2_) is the main pollutant in nuclear waste disposal and the nuclear accident [13,14]. Therefore, acquiring adsorbents for effective capture of I_2_ is on demand.

To date, a broad range of solid adsorbents has been found to be very promising for removing molecular iodine [15,16,17,18,19]. These adsorbents include zeolites [20,21], functionalized clays [22], activated carbon [23], metal/covalent–organic frameworks (MOFs and COFs) [24,25,26,27,28,29,30,31,32,33], supramolecular cages [34,35], supramolecular assemblies [36,37,38], etc. For example, Zheng et al. reported two amorphous MOFs exhibiting very high I_2_ uptake with adsorption capacities of 2.05 and 5.04 g g^−1^ [39], respectively. Chi et al. reported that nonporous adaptive crystals of a bipyridine cage can reversibly capture I_2_ [40]. In spite of that significant progress on adsorbents for I_2_ capture has been achieved, there is still much room to improve the performance of adsorbents for I_2_ capture. In general, a high-performance I_2_ capture material needs to simultaneously meet the following requirements: high I_2_ adsorption capacity and kinetics under industrial conditions, high selectivity, a long retention time of the adsorbed I_2_, and great recyclability and low-cost [14]. The search for high-performance I_2_ capture adsorbents is still ongoing.

Recently, macrocycle-based supramolecular assemblies have emerged as a class of adsorbents for I_2_ capture [37,41,42]. For example, Huang’s group reported perethylated pillar [6] arene, which acts as a candidate for I_2_ capture [41], while Zhang and co-workers directly observed the ambiguous binding sites for I_2_ in a mesoporous assembly of aluminum molecular rings [42]. Recently, we have successfully obtained a series of *π*-stacked porous assemblies based on metal complexes of tripodal tris(2-benzimidazolylmethyl) amine or tris(2-naphthimidazolemethyl) amine [43,44]. These achievements promoted us to synthesize porous assemblies based on metal complexes of tripodal ligands to explore high-performance adsorbents for I_2_ capture.

In this work, we report a porous *π*-stacked self-assembly based on a cup-shaped Pd^II^ complex. Due to the channels in the structure, this material permits the capture of both dissolved I_2_ and I_2_ vapor. Furthermore, the present adsorbent can be reused several times without significant loss of I_2_ uptake capacity.

## 2. Results and Discussion

### 2.1. Structure Characterizations of the π-Stacked Self-Assembly

**Figure 1:**
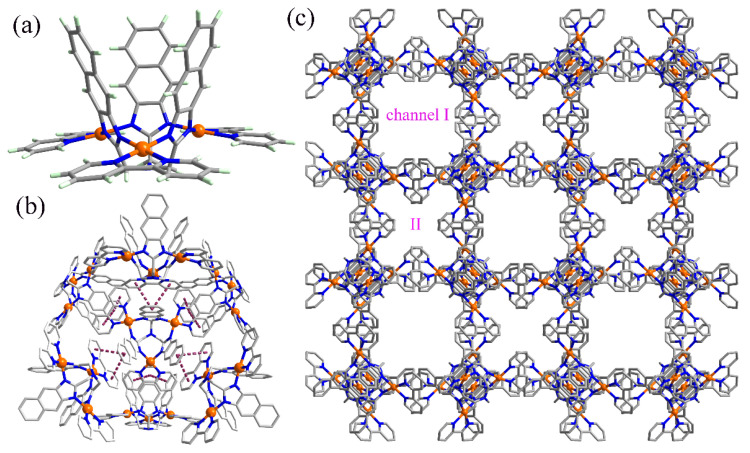
(**a**) The cup-shaped trinuclear [Pd_3_(bipy)_3_L]^3+^ in a macrocycle. Atom color codes: Pd, orange; N, blue; C, gray; H, bright white. (**b**) View of each macrocycle associating with its six neighbors through *π*···*π* interactions. (**c**) The porous cubic supramolecular assembly showing two types of channels.

**Figure 2:**
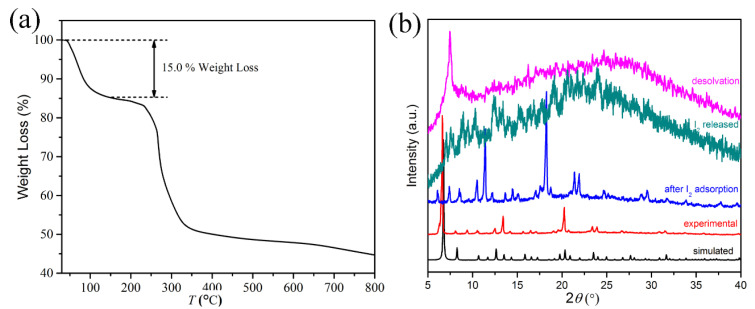
(**a**) TG analysis of compound **1**. (**b**) PXRD patterns of compound **1**.

The self-assembly of (2,2′-bipyridine) dichloropalladium (II)([Pd(bipy)]Cl_2_) with tris(2-naphthimidazolemethyl) amine (H_3_L) in a mixture of MeOH/acetone (*v*/*v*: 1/3) with a trace of triethylamine affords yellow crystals of [Pd_3_(bipy)_3_L] Cl_3_·solvent (**1**). Single-crystal X-ray analysis (Table S1) reveals a cup-shaped trinuclear Pd^II^ complex in which three [Pd(bipy)]^2+^ cations are bridged by the naphthimidazolemethyl arms of L, giving rise to a macrocycle (Figure 1a). Driven by the coordination mentioned above, L is fixed into an unusual cup-shaped conformation [39,40] and the three [Pd(bipy)]^2+^ cations act as the cup holder. In the crystal structure, each [Pd_3_(bipy)_3_L]^3+^ associates with its six neighbors (Figure 1b) through *π*···*π* interactions between bipy and L, forming a porous non-symmetric cubic supramolecular assembly (Figure 1c). This porous structure possesses two kinds of channels along all three crystallographic axes, which are filled with Cl^−^ and solvent molecules. Determined by PLATON, the void volume is 8658 Å^3^ per unit cell, which is 48.3% of the unit volume. In the view of topology, treating [Pd_3_(bipy)_3_L]^3+^ as a node and the *π*···*π* interaction between bipy and L as a linker (Figure S1a), the porous assembly can be simplified as a pcu network with a Schläfli symbol of 4^6^·6^9^ (Figure S1b). Thermogravimetric (TG) analysis with the sample heated under an N_2_ stream revealed a weight loss of ~15% between 30 and 200 °C, which can be attributed to the removal of solvent molecules (Figure 2a). After desolvation, the framework structure of the porous assembly collapses, as indicated by powder X-ray diffraction (PXRD) studies (Figure 2b).

### 2.2. Iodine Adsorption Study

**Figure 3:**
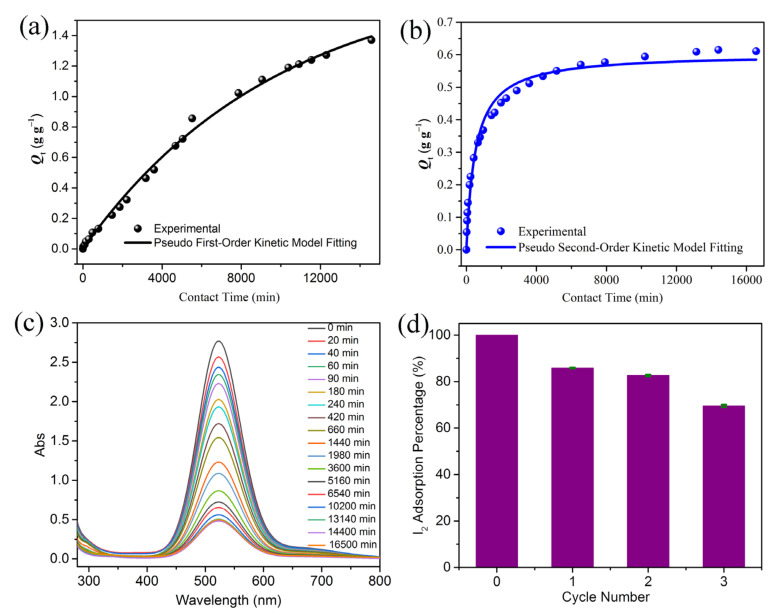
(**a**) Time-dependent iodine vapor uptake plot for the crystals of compound **1** at room temperature. (**b**) Time-dependent dissolved iodine uptake plot for the crystals of compound **1** at room temperature. (**c**) Time-dependent UV–Vis spectrum evolution of the solution of I_2_ in cyclohexane with the crystals of compound **1** as adsorbent. (**d**) Graph showing the recyclability of compound **1** for dissolved iodine adsorption.

The poor thermostability of compound **1** prohibits us from investigating its iodine adsorption performance at high temperatures. Therefore, the adsorption performances of compound **1** on both gaseous and dissolved iodine were investigated at room temperature. Exposing compound **1** to iodine vapor at room temperature led to a gradual color change from yellow to black (Figure S2a). The iodine uptake also gradually increased with time and attained an uptake of 1.37 g g^−1^ after 240 h without saturation (Figure 3a). The gaseous iodine adsorption profile can be well described by the pseudo-first-order kinetic model (*R*^2^ = 0.996), which gives an adsorption rate *k* = 1.0 × 10^−4^ g min^−1^ and an equilibrium adsorption capacity *Q*_e_ = 1.81 g g^−1^ (Table S2).

We then examined the adsorption performance of compound **1** for iodine dissolved in cyclohexane. A crystalline sample of compound **1** (0.05 g) was immersed in a 3 mM iodine–cyclohexane solution. UV–Vis spectroscopy was used to evaluate the iodine adsorption rate (Figure 3b,c and Figure S3). With the adsorption going on, the color of the iodine–cyclohexane solution gradually faded (Figure S2b). The color of the sample of compound **1** gradually deepened and turned black when the adsorption equilibrium was reached (Figure S2c). The monitoring data revealed a fast adsorption rate in the first 6 h, and then the adsorption gradually slowed down until equilibrium (Figure 3b). The experimental data can be well described by the pseudo-second-order kinetic model (*R*^2^ = 0.977), which gives an adsorption rate *k*_2_ = 3.0 × 10^−3^ g min^−1^ and an equilibrium adsorption capacity of 0.60 g g^−1^ (Figure 3b, Table S2). The gaseous I_2_ and dissolved I_2_ uptake capacities of compound **1** are comparable to those of some promising I_2_ adsorbents (Table S3) [45,46,47,48]. Furthermore, the adsorbed iodine can be released from I_2_@**1** by soaking I_2_@**1** in CH_2_Cl_2_. When 0.50 g of solid I_2_@**1** was immersed in CH_2_Cl_2_, the solution gradually changed from colorless to dark brown in 36 h, indicating a large amount of I_2_ was released (Figure S4). Therefore, this adsorbent for iodine capture can be recycled. In the third adsorption–desorption cycle, ~70% of the I_2_ adsorption capability can be retained (Figure 3d).

**Figure 4:**
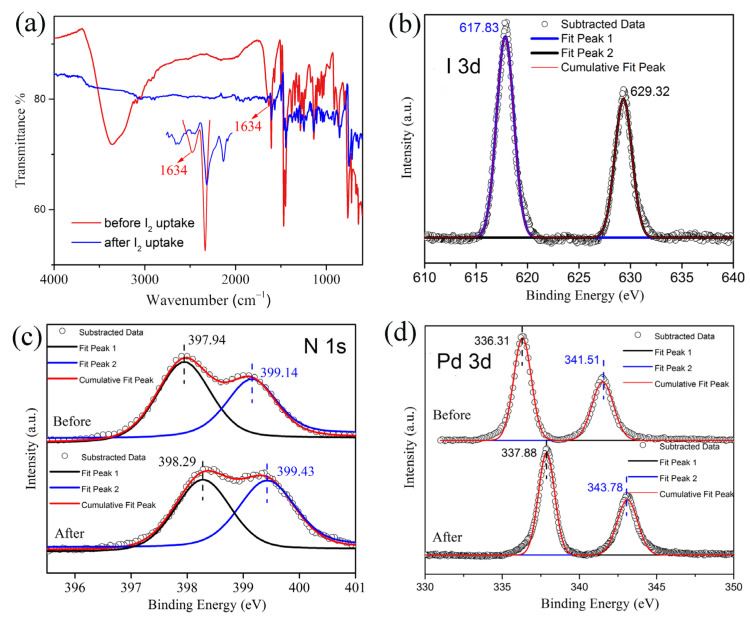
(**a**) IR spectra of compound **1** before and after I_2_ uptake (inset: enlarged spectra showing the significant decrease of band at ∼1640 cm^−1^). (**b**) XPS of I 3d for I_2_@**1**. (**c**) XPS of N 1s for compound **1** before and after dissolved I_2_ uptake. (**d**) XPS of Pd 3d for compound **1** before and after dissolved I_2_ uptake.

To give insights into the I_2_ adsorption mechanism, we conducted Fourier transform infrared (FT-IR) spectroscopy (Figure 4a) and X-ray photoelectron spectroscopy (XPS) (Figure 4b–d) studies on compound **1** before and after I_2_ uptake. After I_2_ loading, the characteristic band at ∼1634 cm^−1^ assigned to the C=N stretching vibration decreases significantly [14,19,29,33,34]. A pair of I 3d signals can be seen from the XPS of the sample after I_2_ uptake (Figure 4a,b). The signals at 617.84 and 629.37 eV can be attributed to I 3d_5/2_ and I 3d_3/2_, respectively. After I_2_ loading, the two N 1s signals shift from 397.94 and 399.14 eV to 398.29 and 399.43 eV, respectively (Figure 4c). The two Pd 3d signals also shift from 336.31 and 341.51 eV to 337.88 and 343.78 eV, respectively (Figure 4d). These results indicate that the N and Pd atoms on compound **1** interact with the captured iodine [49]. This interaction may be rationalized in terms of that polarized bound iodine molecules favor interaction with the partly negatively charged N lone pairs, while the cylindrical electron surface of the I−I bond would favor interaction with the positively charged Pd atoms [45]. The PXRD of I_2_@**1** is significantly different from that of compound **1,** indicating a possible significant structural change upon iodine adsorption. However, the poor crystallinity of I_2_@**1** prohibits us from directly observing the I_2_ binding sites by single-crystal X-ray analysis. The recycled sample of compound **1** that lost crystallinity probably implies good dispersion of the adsorbed iodine molecules around the cup-shaped molecules (Figure 2b).

## 3. Experimental

### 3.1. Iodine Adsorption Study

The ligand H_3_L is synthesized according to the previously reported method [50]. [Pd(bipy)] Cl_2_ and 2,3-diaminonaphthalene were purchased from bidepharmatech. All other reagents were purchased from Adamas (Shanghai, China) and used directly, without purification.

### 3.2. Characterization

Fourier-transform infrared (FTIR, Nicolet iS 50, Thermo Fisher, Waltham, MA, USA) spectra were recorded on a Thermo Fisher Nicolet iS 50 in the range 500–4000 cm^−1^ at room temperature. Powder X-ray diffraction (PXRD, Miniflex 600, Akishima, Rigaku, Tokyo, Japan) patterns were obtained on a Miniflex 600 diffractometer using Cu-Kα radiation with flat plate geometry. X-ray photoelectron spectroscopy (XPS, Thermo Scientific K-Alpha, Waltham, MA, USA) studies were performed on an AXIS SUPRA Kratos system, and the C 1s line at 284.8 eV was used as the binding energy reference. TGA was performed using a thermo plus EVO2 system at a rate of 10 °C/min in the range of 30–800 °C (TGA/DSC 1, Mettler Telodo, Zurich, Switzerland). UV–Vis spectra were recorded on an Agilent Cary 5000 spectrophotometer (UV-Vis, Agilent, Santa Clara, CA, USA). 

### 3.3. Crystallography

Single-crystal X-ray data were harvested on a Bruker D8 Venture diffractometer with Mo-Kα radiation at 200 K. Structures were solved using a direct method and refined by the full-matrix least-squares technique on F2 with the SHELXTL 2014 program [51]. All the H atoms are geometrically generated and refined using a riding model. The PLATON/SQUEEZE procedures [52] were used to treat the highly disordered solvents in the void of the porous structure. The X-ray crystallographic coordinates for structures reported in this article have been deposited at the Cambridge Crystallographic Data Centre (CCDC), under deposition number CCDC 2245193. The data can be obtained free of charge from the Cambridge Crystallographic Data Centre via www.ccdc.cam.ac.uk/data_request/cif (accessed on 28 February 2023). Details of the crystallographic data are listed in Table S1.

### 3.4. Synthesis of Compound **1**

Additionally, (2,2′-bipyridine) dichloropalladium (II) (0.030 g, 0.09 mmol), tris (2-naphthimidazole methyl) amine (H_3_L) (0.020 g, 0.036 mmol), and triethylamine (0.02 mL) were added to a mixture of MeOH/acetone (*v*/*v*: 1/3), and the mixture was stirred at room temperature for 2 h. After that, the insoluble substance was removed through filtration. The resulting filtrate was kept at room temperature undisturbed for 7 days, and then pale green crystals were obtained (yield: 67.7% based on L).

### 3.5. Iodine Adsorption Experiments

Both the gaseous iodine and dissolved iodine uptake behaviors of compound **1** were studied at room temperature.

#### 3.5.1. Iodine Vapor Adsorption

Air-dried compound 1 (0.050 g) was loaded into an uncapped glass vial, which was located in a sealed container with excess solid iodine kept at the bottom. After certain time intervals, the vial was taken out and weighed, and then reloaded into the vapor of iodine to continue adsorption. The iodine uptake at a certain time was calculated using Equation (1):

(1) $$Q_{t}=\frac{m_{2}-m_{1}}{m_{1}}$$

where *Q_t_* represents the iodine uptake at a certain time and *m*_1_ and *m*_2_ are the masses of the sample of compound **1** before and after iodine uptake, respectively. The pseudo-first-order model (Equation (2)) was used to fit the gaseous iodine adsorption profile, giving a set of parameters with *k*_1_ = 1.0 × 10^−4^ g min^−1^, *Q_e_* = 1.81 g g^−1^, and *R^2^* = 0.996.

(2) $$Q_{t}=Q_{e}\left(1-e^{k_{1}t}\right)$$

#### 3.5.2. Iodine Adsorption in Solution

Air-dried compound **1** (0.050 g) was immersed in a 50 mL solution of iodine in cyclohexane (3 mM). The iodine adsorption process was monitored by UV–Vis spectroscopy. The iodine uptake was calculated using Equation (3):

(3) $$Q_{t}=\frac{\left(C_{0}-C_{t}\right)}{m}V$$

where *Q_t_* represents the iodine uptake at a certain time, *C*_0_ and *C_t_* represent the concentration of iodine before and after adsorption, respectively, *m* represents the mass of compound **1**, and *V* represents the volume of the solution. The pseudo-second-order model (Equation (4)) was used to fit the dissolved iodine adsorption profile, giving a set of parameters with *k*_2_ = 3.0 × 10^−3^ g min^−1^, *Q_e_* = 0.60 g g^−1^, and *R*^2^ = 0.977.

(4) $$Q_{t}=\frac{k_{2}Q_{e}^{2}t\;}{1+k_{2}Q_{e}t}$$

#### 3.5.3. Iodine Release and Recyclability of Compound **1**

I_2_@**1** was immersed in CH_2_Cl_2_ to release the adsorbed iodine. Here, I_2_@**1** (0.050 g) was immersed in CH_2_Cl_2_ (100 mL). When the release was deemed essentially complete, the resulting solid was recycled and analyzed by PXRD. Then the recycled solid of compound **1** was added to the I_2_/cyclohexane solution again. After four cycles, ~70% of the I_2_ adsorption capability can be retained.

## 4. Conclusions

In summary, we have developed a porous self-assembly of a cup-shaped Pd^II^ complex. This porous structure is constructed through intermolecular *π*···*π* interactions. The channels along all three crystallographic axes within the self-assembly allow for efficient reversible iodine capture, either from the vapor or solution source phases. These results demonstrate that porous crystalline materials assembled through weak intermolecular interactions can serve as a new type of promising adsorbent for I_2_ capture.

## Data Availability

All data related to this study are presented in this publication.

## Supplementary Materials

The following supporting information can be downloaded at: https://www.mdpi.com/article/10.3390/molecules28072881/s1, Figure S1: (a) The simplification of the network of compound **1**, (b) The simplified pcu network of the supramolecular framework of compound 1; Table S1: Crystallographic data of compound **1**; Figure S2: (a) The setup for I_2_ vapor adsorption, (b) Photographs showing color changes of the I_2_/cyclohexane solution as a function of time when 0.050 g of compound **1** was immersed in the solution, (c) Photographs showing the color change of the crystals of compound **1** before and after dissolved I_2_ adsorption, (d) Photographs showing the release of I_2_ from I_2_@**1** in CH_2_Cl_2_; Table S2: Fitting the iodine adsorption kinetics of compound **1**; Figure S3: Standard plot between absorbance (λ = 523 nm) and I_2_ concentration of the solution of I_2_ in cyclohexane; Table S3: The comparison of I_2_ adsorption capacities for various adsorbents.

## Sample Availability

Samples are available from the author.

## References

[1] Xie W., Cui D., Zhang S.-R., Xu Y.-H., Jiang D.-L. (2019). Iodine capture in porous organic polymers and metal∓organic frameworks materials. Mater. Horiz..

[2] Ewing R.C., Hippel F.N.V. (2009). Nuclear waste management in the United States-starting over. Science.

[3] Sen A., Sharma S., Dutta S., Shirolkar M.M., Dam G.K., Let S., Ghosh S.K. (2021). Functionalized ionic porous organic polymers exhibiting high iodine uptake from both the vapor and aqueous medium. ACS. Appl. Mater. Interfaces.

[4] Yan Z.J., Qiao Y.M., Wang J.L., Xie J.L., Cui B., Fu Y., Lu J.W., Yang Y.J., Bu N.S., Yuan Y. (2022). An azo-group-functionalized porous aromatic framework for achieving highly efficient capture of iodine. Molecules.

[5] Kintisch E. (2005). Congress tells DOE to take fresh look at recycling spent reactor fuel. Science.

[6] Ogilvy-Stuart A.L., Shalet S.M. (1993). Effect of radiation on the human reproductive system. Environ. Health Perspect..

[7] Chen P., He X.H., Pang M.B., Dong X.T., Zhao S., Zhang W. (2020). Iodine capture using Zr-based metal–organic frameworks (Zr-MOFs): Adsorption performance and mechanism. ACS. Appl. Mater. Interfaces.

[8] Gao R., An B.H., Zhou C., Zhang X. (2022). Synthesis of a triazaisotruxene-based porous organic polymer and its application in iodine capture. Molecules.

[9] Shimamoto Y.S., Takahashi Y., Terada Y. (2011). Formation of organic iodine supplied as iodide in a soil-water system in Chiba, Japan. Environ. Sci. Technol..

[10] Sabri M.A., Al-Sayah M.H., Sen S., Ibrahim T.H., El-Kadri O.M. (2020). Fluorescent aminal linked porous organic polymer for reversible iodine capture and sensing. Sci. Rep..

[11] Ten Hoeve J.E., Jacobson M.Z. (2012). Worldwide health effects of the fukushima daiichi nuclear accident. Energy Environ. Sci..

[12] Taylor D.M. (1981). The radiotoxicology of iodine. J. Radioanal. Chem..

[13] Yamaguchi N., Nakano M., Takamatsu R., Tanida H. (2010). Inorganic iodine incorporation into soil organic matter: Evidence from iodine K-edge X-ray absorption near-edge structure. J. Environ. Radioact..

[14] Yang Y.T., Tu C.Z., Yin H.J., Liu J.J., Cheng F.X., Luo F. (2022). Molecular iodine capture by covalent organic frameworks. Molecules.

[15] Yu C.-X., Li X.-J., Zong J.-S., You D.-J., Liang A.-P., Zhou Y.-L., Li X.-Q., Liu L.-L. (2022). Fabrication of protonated two-dimensional metal–organic framework nanosheets for highly efficient iodine capture from water. Inorg. Chem..

[16] Zhang X.R., Maddock J., Nenoff T.M., Denecke M.A., Yang S., Schröder M. (2022). Adsorption of iodine in metal–organic framework materials. Chem. Soc. Rev..

[17] Yan Z.J., Cui B., Zhao T., Luo Y.F., Zhang H.C., Xie J.L., Li N., Bu N.S., Yuan Y., Xia L.X. (2021). A carbazole-functionalized porous aromatic framework for enhancing volatile iodine capture via lewis electron pairing. Molecules.

[18] Guan H., Zou D.L., Yu H.Y., Liu M.J., Liu Z., Sun W.T., Xu F.F., Li Y.X. (2019). Adsorption behavior of iodine by novel covalent organic polymers constructed through heterostructural mixed linkers. Front. Mater..

[19] Tian P., Ai Z.T., Hu H., Wang M., Li Y.L., Gao X.P., Qian J.Y., Su X.F., Xiao S.T., Xu H.J. (2022). Synthesis of electron-rich porous organic polymers via schiff-base chemistry for efficient iodine capture. Molecules.

[20] Pham T.C.T., Docao S., Hwang I.C., Song M.K., Choi D.Y., Moon D., Oleynikov P., Yoon K.B. (2016). Capture of iodine and organic iodides using silica zeolites and the semiconductor behaviour of iodine in a silica zeolite. Energy Environ. Sci..

[21] Chapman K.W., Chupas P.J., Nenoff T.M. (2010). Radioactive iodine capture in silver-containing mordenites through nanoscale silver iodide formation. J. Am. Chem. Soc..

[22] Reda A.T., Zhang D.X., Xu X.Y., Xu S.Y. (2022). Highly stable iodine capture by pillared montmorillonite functionalized Bi2O3@g-C3N4 nanosheets. Sep. Purif. Technol..

[23] Deuber H. (2017). Investigations on the retention of elemental radioiodine by activated carbons at high temperatures. Nucl. Technol..

[24] Zhang Y.B., Cui X.L., Xing H.B. (2021). Recent advances in the capture and abatement of toxic gases and vapors by metal–organic frameworks. Mater. Chem. Front..

[25] Mondal S., Dastidar P. (2019). Mixed ligand cordination polymers for metallogelation and iodine adsorption. Cryst. Growth Des..

[26] Song S.N., Shi Y., Liu N., Liu F.Q. (2021). Theoretical screening and experimental synthesis of ultrahigh-iodine capture covalent organic frameworks. ACS. Appl. Mater. Interfaces.

[27] Wang C., Wang Y., Ge R., Song X.D., Xing X.Q., Jiang Q.K., Lu H., Hao C., Guo X.W., Gao Y.N. (2018). A 3D covalent organic framework with exceptionally high iodine capture capability. Chem. Eur. J..

[28] Yin Z.-J., Xu S.-Q., Zhan T.-G., Qi Q.-Y., Wu Z.-Q., Zhao X. (2017). Ultrahigh volatile iodine uptake by hollow microspheres formed from a heteropore covalent organic framework. Chem. Commun..

[29] Yan X., Yang Y.X., Li G.R., Zhang J.H., He Y., Wang R., Lin Z., Cai Z.W. (2023). Thiophene-based covalent organic frameworks for highly efficient iodine capture. Chin. Chem. Lett..

[30] Zaguzin A.S., Sukhikh T.S., Kolesov B.A., Sokolov M.N., Fedin V.P., Adonin S.A. (2022). Lodinated vs non-iodinated: Comparison of sorption selectivity by [Zn_2_(bdc)_2_dabco]n and superstructural 2-iodoterephtalate-based metal–organic framework. Polyhedron.

[31] Zaguzin A.S., Mahmoudi G., Sukhikh T.S., Sakhapov I.F., Zherebtsov D.A., Zubkov F.I., Valchuk K.S., Sokolov M.N., Fedin V.P., Adonin S.A. (2022). 2D and 3D Zn (II) coordination polymers based on 4′-(Thiophen-2-yl)-4,2′:6′,4′’-terpyridine: Structures and features of sorption behavior. J. Mol. Struct..

[32] Yadollahi M., Hamadi H., Nobakht V. (2020). Capture of iodine in solution and vapor phases by newly synthesized and characterized encapsulated Cu_2_O nanoparticles into the TMU-17-NH2 MOF. J. Hazard. Mater..

[33] Li H.L., Cheng D.S., Cheng Z.K., Li Z.Y., Li P.-Z. (2023). Effective iodine adsorption by nitrogen-rich nanoporous covalent organic frameworks. ACS Appl. Nano Mater..

[34] Hasell T., Schmidtmann M., Cooper A.I. (2011). Molecular doping of porous organic cages. J. Am. Chem. Soc..

[35] Liu C., Li W.L., Liu Y., Wang H.L., Yu B.Q., Bao Z.B., Jiang J.Z. (2022). Porous organic cages for efficient gas selective separation and iodine capture. Chem. Eng. J..

[36] Chen X.Y., Zhang T., Han Y.N., Chen Q., Li C.P., Xue P.C. (2021). Multi-responsive fluorescent switches and iodine capture of porous hydrogen-bonded self-assemblies. J. Mater. Chem. C..

[37] Li B., Wang B., Huang X.Y., Dai L., Cui L., Li J., Jia X.S., Li C.J. (2019). Terphen[n]arenes and quaterphen[n]arenes (n = 3–6): One-pot synthesis, self-assembly into supramolecular gels, and iodine capture. Angew. Chem. Int. Ed..

[38] Luo D., Wang F., Liu C.-H., Wang S.-T., Sun Y.-Y., Fang W.-H., Zhang J. (2022). Combination of aluminum molecular rings with chemical reduction centers for iodine capture and aggregation. Inorg. Chem. Front..

[39] Feng Y., Zou M.-Y., Hu H.-C., Li W.-H., Cai S.-L., Zhang W.-G., Zheng S.-R. (2022). Amorphous metal-organic frameworks obtained from a crystalline precursor for the capture of iodine with high capacities. Chem. Commun..

[40] Luo D., He Y.L., Tian J.Y., Sessler J.L., Chi X.D. (2022). Reversible iodine capture by nonporous adaptive crystals of a bipyridine cage. J. Am. Chem. Soc..

[41] Jie K.C., Zhou Y.J., Li E.E., Li Z.T., Zhao R., Huang F.H. (2017). Reversible iodine capture by nonporous pillar[6]arene crystals. J. Am. Chem. Soc..

[42] Yao S.Y., Fang W.-H., Sun Y.Y., Wang S.-T., Zhang J. (2021). Mesoporous assembly of aluminum molecular rings for iodine capture. J. Am. Chem. Soc..

[43] Li G.L., Zhuo Z., Wang B., Cao X.L., Su H.F., Wang W., Huang Y.G., Hong M.C. (2021). Constructing π-stacked supramolecular cage based hierarchical self-assemblies via π···π stacking and hydrogen bonding. J. Am. Chem. Soc..

[44] Li S., Li G.-L., Wang W., Liu Y., Cao Z.-M., Cao X.-L., Huang Y.-G. (2021). A 2D metal-organic framework interpenetrated by a 2D supramolecular framework assembled by CH/π interactions. Inorg. Chem. Commun..

[45] Zhao Q., Zhu L., Lin G.H., Chen G.Y., Liu B., Zhang L., Duan T., Lei J.H. (2019). Controllable synthesis of porous Cu-BTC@polymer composite beads for iodine capture. ACS. Appl. Mater. Interfaces.

[46] Wang L.Y., Li T., Dong X.T., Pang M.B., Xiao S.T., Zhang W. (2021). Hiophene-based MOFs for iodine capture: Effect of pore structures and interaction mechanism. Chem. Eng. J..

[47] Pan X.W., Ding C.H., Zhang Z.M., Ke H.Z., Cheng G. (2020). Functional porous organic polymer with high S and N for reversible iodine capture. Microporous. Mesoporous. Mater..

[48] Mahdi E.M., Chaudhuri A.K., Tan J.C. (2016). Capture and immobilisation of iodine (I_2_) utilising polymer-based ZIF-8 nanocomposite membranes. Mol. Syst. Des. Eng..

[49] Kim H., Doan V.D., Cho W.J., Madhav M.V., Kim K.S. (2014). Anisotropic charge distribution and anisotropic van der waals radius leading to intriguing anisotropic noncovalent interactions. Sci. Rep..

[50] Rodionov V.O., Presolski S.I., Gardinier S., Lim Y.H., Finn M.G. (2007). Benzimidazole and related ligands for Cu-catalyzed azide-alkyne cycloaddition. J. Am. Chem. Soc..

[51] Sheldrick G.M. (2015). SHELXTL-integrated space-group and crystal-structure determination. Acta Crystallogr..

[52] Spek A.L. (2003). Single-crystal structure validation with the program PLATON. J. Appl. Crystallogr..

