# Supplementary material for: A Porous π-Stacked Self-Assembly of Cup-Shaped Palladium Complex for Iodine Capture
Source: Molecules. 2023 Mar 23;28(7):2881. doi: 10.3390/molecules28072881 (PMC10095786; doi:10.3390/molecules28072881)
Supplement: Supplementary file 1 [file molecules-28-02881-s001.zip › Supporting Information.pdf]

A Porous π-Stacked Self-Assembly of Cup-Shaped Palladium Complex for Iodine Capture

Lin-Lin Li[1,4,5], Min Huang[3,4,5], Ting Chen[3,4,5], Xiao-Feng Xu[4,5], Zhu Zhuo[4,5], Wei Wang[4,5], You-Gui Huang[2,4,5*]

[1] College of Chemistry, Fuzhou University, Fuzhou, 350108, China
[2] Fujian Science & Technology Innovation Laboratory for Optoelectronic Information of China, Fuzhou, Fujian, 350108, China
[3] College of Chemistry and Materials Science, Fujian Normal University, Fuzhou, Fujian, 350002, P. R. China
[4] CAS Key Laboratory of Design and Assembly of Functional Nanostructures, and Fujian Provincial Key Laboratory of Nanomaterials, Fujian Institute of Research on the Structure of Matter, Chinese Academy of Sciences, China
[5] Xiamen Institute of Rare Earth Materials, Haixi Institute, Chinese Academy of Science, Xiamen 361021, China

Table S1. Crystallographic data of compound **1**.

Compound	**1**
Formula	$Pd_3C_{66}H_{48}N_{13}$
Formula weight	1342.37
Temp. (*K*)	200
Crystal System	Cubic
Space group	I–43m
a (Å)	26.0030(2)
V(Å^3)	17582.1(4)
Z	8
$\rho_{cal.}$(g cm^{-3})	1.014
μ	0.643
F(000)	5384
θ *range* (°)	4.43–67.346
Reflections ($I > 2\sigma$)	5662
$R_1(I > 2\sigma)$	0.0459
wR_2(*all*)	0.1342
GOF on F^2	1.083
CCDC#	2245193

[a]$R_1 = \Sigma||F_o| - |F|| / \Sigma|F_o|$ and $wR_2 = [\Sigma w(F_o^2 - F_c^2)^2 / \Sigma wF_o^4]^{1/2}$ for $F_o^2 > 2\sigma(F_o^2)$

Table S2. Fitting the iodine adsorption kinetics of compound **1**.

adsorbate	I_2 vapor			Dissolved I_2		
model	pseudo first-order			pseudo second-order		
parameter	k_1 (g g^{-1} min^{-1})	Q_e (g g^{-1})	R^2	k_2 (g g^{-1} min^{-1})	Q_e (g g^{-1})	R^2
	1.0×10^{-4}	1.81	0.996	3.0×10^{-3}	0.603	0.977

Table S3. The comparison of I_2 adsorption capacities for various adsorbents.

Adsorbents	I_2 vapor (g g^{-1})	Adsorption of I_2 in solution (g g^{-1})	Ref.
Cu-BTC@PES	0.639	0.260	41
DUT-68	1.08	/	42
P-TzTz	/	0.494	43
ZIF-8@PU	/	0.33	44
**1**	1.81	0.60	This work

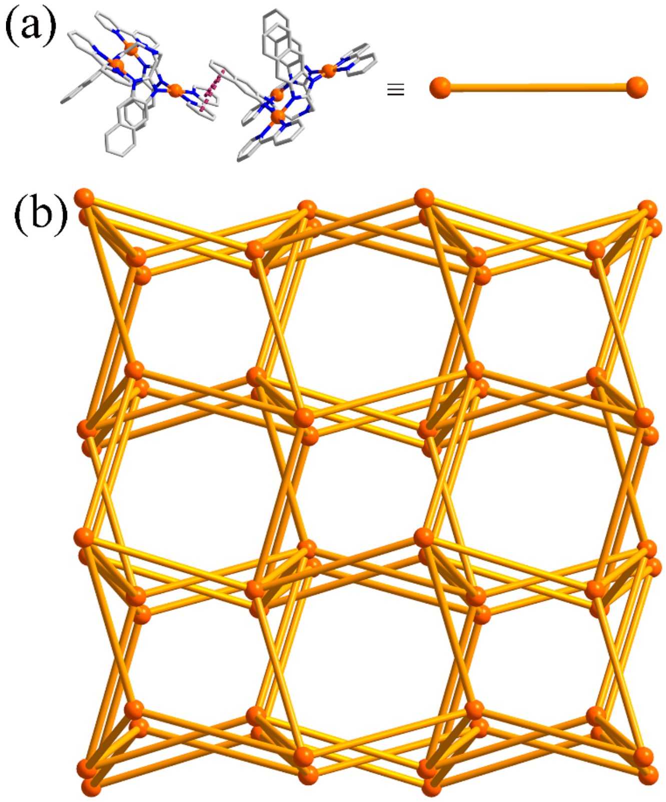

Figure S1. (a) The simplification of the network of compound **1**. (b) The simplified **pcu** network of the supramolecular framework of compound **1**.

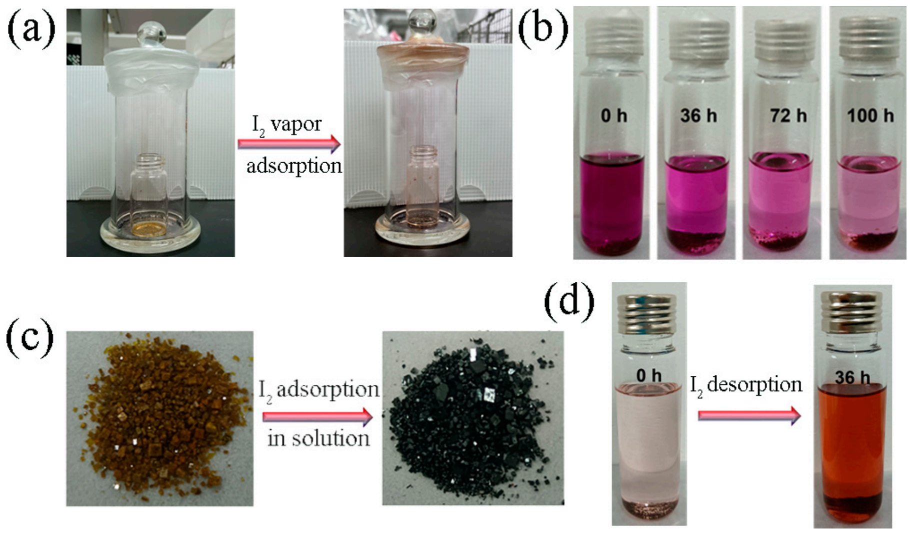

Figure S2. (a) The setup for I_2 vapor adsorption. (b) Photographs showing color changes of the I_2/cyclohexane solution as a function of time when 0.050 g compound **1** was immersed in the solution. (c) Photographs showing the color change of the crystals of compound **1** before and after dissolved I_2 adsorption. (d) Photographs showing the release of I_2 from I_2@**1** in CH_2Cl_2.

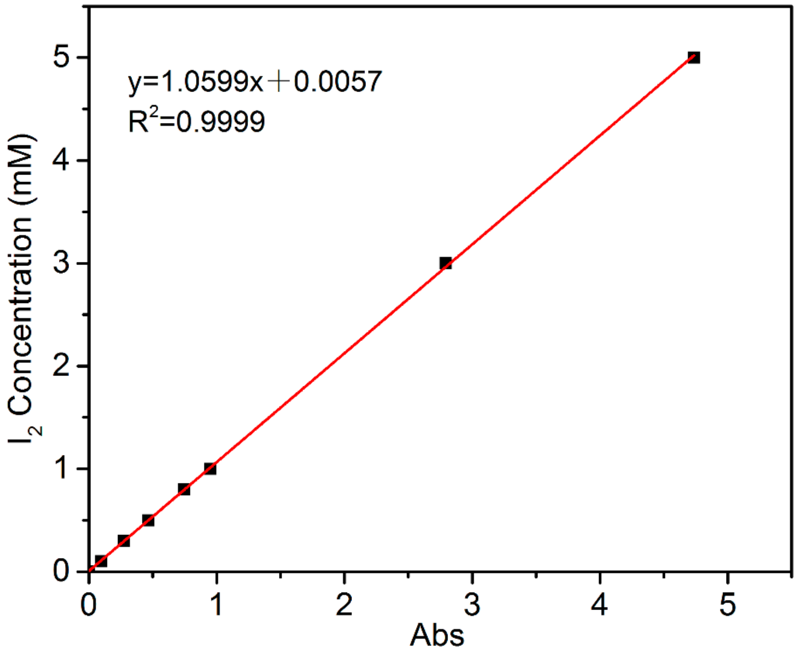

Figure S3. Standard plot between absorbance (λ = 523 nm) and I_2 concentration of the solution of I_2 in cyclohexane.
